# Antimicrobial knowledge and confidence amongst final year medical students in Australia

**DOI:** 10.1371/journal.pone.0182460

**Published:** 2017-08-03

**Authors:** Naomi Weier, Karin Thursky, Syed Tabish R. Zaidi

**Affiliations:** 1 Division of Pharmacy, School of Medicine, University of Tasmania, Hobart, Tasmania, Australia; 2 Doherty Institute, Melbourne, Victoria, Australia; 3 The Royal Melbourne Hospital, Parkville, Victoria, Australia; National Institute of Health, ITALY

## Abstract

**Introduction:**

Inappropriate use of antimicrobials is one of the major modifiable contributors to antimicrobial resistance. There is currently no validated survey tool available to assess knowledge and confidence of medical students in infectious diseases (ID) compared to other diseases states, and little is known about this topic.

**Materials and methods:**

A cross-sectional survey of final year medical students attending universities around Australia was conducted between August and September, 2015. A survey unique from other published studies was developed to survey satisfaction in education, confidence and knowledge in ID, and how this compared to these factors in cardiovascular diseases.

**Results:**

Reliability and validity was demonstrated in the survey tool used. Students were more likely to rate university education as sufficient for cardiovascular diseases (91.3%) compared to ID (72.5%), and were more confident in their knowledge of cardiovascular diseases compared to ID (74.38% vs. 53.76%). Students tended to answer more cardiovascular disease related clinical questions correctly (mean score 78%), compared to questions on antimicrobial use (mean score 45%).

**Conclusions:**

Poor knowledge and confidence amongst final year medical students in Australia were observed in ID. Antimicrobial stewardship agenda should include the provision of additional training in antimicrobial prescribing to the future medical workforce.

## Introduction

Inappropriate antimicrobial prescribing amongst medical practitioners with a broad range of expertise and experience is highly prevalent, and is associated with increasing antimicrobial resistance [1–7]. In Australia, both smaller studies and nation-wide research has shown that there is a high incidence of inappropriate antimicrobial prescribing and use [4,6,7]. The most recent Australia National Antimicrobial Prescribing Survey report found that inappropriate antimicrobial prescribing was observed in 24.4% of all prescriptions [6]. An American survey study found no difference in the knowledge and use of antimicrobials between physicians who were at different stages of their professional careers [8]. This suggests that medical practitioners in all areas and with varying experiences can struggle with appropriate antimicrobial prescribing in clinical practice.

Traditionally, education on appropriate antimicrobial prescribing has been aimed at medical practitioners after they have completed their university education and training, however once prescribing habits are formed they can be difficult to change [9,10]. Australian research has found that there are multiple influences that affect the prescribing of antimicrobials in clinical practice, including professional hierarchies, reputation, and pressures to prescribe antimicrobials based on the acceptable clinical practice amongst peers instead of according to guidelines [11]. It is, therefore, essential that medical graduates are equipped with adequate knowledge and skills in appropriate antimicrobial prescribing prior to their entry into the medical workforce. One Australian study of prescribing errors made by recent graduates at a major teaching hospital found that almost 25% of errors were related to antimicrobial treatment, highlighting the need for further training and education in antimicrobial use amongst medical graduates [12].

There is an increasing interest in measuring medical students’ knowledge and confidence of ID, antimicrobial prescribing, and resistance [13–15]. A recent European survey of final year medical students found a low level of confidence in the selection of appropriate antibiotic treatment, the right dose and dosing interval for antibiotic treatment, knowing when not to use combination antibiotics, and when not to prescribe antibiotics [15]. The majority of surveyed students wanted more education about infectious diseases as part of their medical training [15]. Two independent survey studies of medical students in the United States echoed the findings of the European study, where students felt they would benefit from additional education on infectious diseases and appropriate antimicrobial use as a part of their university education [13,14].

To the best of our beliefs, no survey study of medical students has utilised a control construct to compare and contrast ID knowledge with the knowledge of non-infectious diseases components of the medical curriculum. Therefore, it is unknown if the surveyed students lack the overall knowledge of medicine in general, or the lack of knowledge and understanding was in the area of ID in particular. Little is also known of the knowledge and confidence of Australian medical students in infectious diseases and antimicrobial use. Finally, other survey tools used in previous studies of medical students did not report reliability or validity of the survey tool [13–15], so there currently is no reliable survey tool available in the literature for this subject matter. As such, the aims of this study were to develop a validated and reliable survey tool in this subject area, and determine the confidence and levels of knowledge of Australian final year medical students in managing infectious diseases and appropriate antimicrobial prescribing as well as compare the knowledge of ID with the knowledge of cardiology.

## Materials and methods

An online survey of final year medical students around Australia was conducted over a period of eight weeks from August to September 2015. The survey tool consisted of six sections on: demographics relating to age, state of study and whether an undergraduate or graduate medical degree was being undertaken; formal education and training at university; confidence in antibiotic prescribing; knowledge and attitudes towards antimicrobial prescribing guidelines; perceptions of antimicrobial resistance; and clinical cases to assess knowledge of antimicrobial prescribing. Participation in this survey was completely voluntary, and by completing the survey this was taken as implied consent. The survey tool is available as S1 Appendix.

### Survey development

An item pool of forty questions was generated based on a thorough literature review [11,12–16], through a consensus approach with experts in infectious diseases and antimicrobial stewardship (STRZ and KAT), and a critical review of the available survey tools [13–15]. The tool was also reviewed by the clinical dean of Melbourne University Medical School. S1 Appendix provides details of the development of the survey.

Following the initial design of the survey based on surveys used in other studies and the inclusion of questions relevant to our research, the draft survey was circulated to a pharmacy academic, infectious diseases physician and Dean of an Australian medical school for review and comments. Any suggestions and comments made by these academics to ensure the validity of the survey to target participants were incorporated into the survey and then recirculated for final approval. Several final year medical students were involved in the initial piloting of the survey to assess understanding and validity before it was made available to all target participants.

### Survey deployment

A stratified random sample of medical schools based on geographical distribution was drawn up from the national list of medical schools, and included medical schools from all states and territories that offered a medical degree, as well as undergraduate and graduate degree students [17].

### Recruitment

Secondary to privacy concerns, we were unable to make a direct contact with the participants and relied on individual universities to pass on the information to the potential participants. In order to ensure responses were representative of students around Australia, universities from every state were approached and invited to participate via telephone and email.

A total of eight universities agreed to share this study with their students. An invitation to participate with a link to the online survey and an information sheet was emailed to the contact person at each university, with a request that this be passed on to their final year medical students. Reminder emails were sent to the university contact person every two weeks while the survey was open. In order to maximise response rate and promote the survey to all final year students at the individual universities, an email was also sent to the student organisations of the medical schools, explaining the aims of the study and a request to pass on the invitation to their members. Participation in the survey was anonymous.

An incentive to win one of eight $100 gift card vouchers was also used to maximise the response rate. The study received ethics approval from the University of Tasmania Social Sciences Human Research Ethics Committee (Reference No.: H0014862).

### Data analysis

Data collected from the survey was analysed using IBM SPSS Statistics Version 23. Cronbach’s alpha was used to assess the reliability of the survey tool. Pearson correlation was used to determine the criterion-related validity of the survey tool [18]. The one sample binomial test was used to determine whether responses between student groups were significantly different, while the Mann-Whitney U test was used to compare differences between student groups. Answers to questions in the clinical cases were grouped based on subject area (infectious diseases and cardiology), and compared collectively.

## Results

### Survey reliability

As this survey was used for the first time in this study, reliability analysis of the survey tool was undertaken.

Cronbach’s alpha >0.7 (used to determine reliability) [19] was obtained for questions relating to sufficiency of education, confidence in knowledge in different subject areas, confidence in different clinical situations, and perceptions of antimicrobial resistance. Item total statistics and inter-item correlation matrix were calculated for different sections of the survey. With the exception of two items and the level of impact they were believed to contribute to antimicrobial resistance (few antibiotics being developed and not prescribing antibiotics when the situation requires its use), all factors exhibited no significant correlation, demonstrating independence from each other. S2 Appendix provides details of this analysis.

### Participants and response rate

191 responses were received from the students and while we are unable to estimate a robust response rate, a representative sample was achieved from all states except Victoria (Table 1). 163 responses were used for analysis as 28 responses were excluded due to only providing demographic information. Participants provided a good representation of the different demographics of Australian medical students (Table 1).

**Table 1:** Reported confidence levels in ID based on different variables (N = 163).

Study Variable	Confident or Most Confident Cohort^1^ N = 86	Somewhat Confident Cohort^1^ N = 73
**Age**		
<25 years old (n = 81, 50.6%)	53 (65.4%)	28 (34.6%)
25–30 (n = 59, 36.9%)	27 (46.6%)	31 (53.4%)
31–35 (n = 11, 6.9%)	3 (27.3%)	8 (72.7%)
36+ (n = 9, 5.6%)	3 (33.3%)	6 (66.7%)
Female (n = 93, 58.1%)	40 (43%)	51 (54.8%)
Undergraduate Degree (n = 97, 59.5%)	59 (60.8%)	35 (36.1%)
State of study		
ACT (n = 6, 3.8%)	3 (50%)	3 (50%)
NSW (n = 27, 16.9%)	15 (55.6%)	12 (44.4%)
Qld (n = 38, 23.4%)	28 (73.7%)	10 (26.3%)
SA (n = 43, 26.9%)	21 (48.8%)	22 (51.2%)
Tas (n = 23, 14.4%)	10 (43.5%)	13 (56.5%)
WA (n = 10, 6.3%)	7 (70%)	2 (20%)
Vic (n = 7, 4.4%)	2 (28.6%)	5 (71.4%)
Other (n = 6, 3.8%)	0	6 (100%)
Sufficient/more than sufficient education in ID^2^ (n = 116, 72.5%)	77 (67%)	38 (33%)
Insufficient education in ID^2^ (n = 44, 27.5%)	9 (20.5%)	35 (79.5%)
Confident/most confident in cardiovascular diseases (n = 118, 74.2%)^3^	81 (68.6%)	37 (31.4%)
Sufficient/more than sufficient education in cardiovascular diseases (n = 146, 91.3%)^4^	84 (57.5%)	61 (41.8%)

NB: Some totals may not equal 100% as some students chose not to answer all questions

^1^Students were asked to report their self confidence level about knowledge of ID and antibiotic prescribing on a scale of 1–3, where 1 = somewhat confident, 2 = confident, 3 = most confident

^2^Students were asked to report the sufficiency of education they received at university in ID and antibiotic prescribing on a scale of 1–3, where 1 = not sufficient, 2 = sufficient, 3 = more than sufficient

^3^Students were asked to report their self confidence levels about knowledge of cardiovascular diseases on a scale of 1–3, where 1 = somewhat confident, 2 = confident, 3 = most confident

^4^Students were asked to report the sufficiency of education they received at university in cardiovascular diseases on a scale of 1–3, where 1 = not sufficient, 2 = sufficient, 3 = more than sufficient

### Sufficiency of education and confidence in knowledge

Relatively more students reported their education at university in cardiology to be ‘sufficient’ or ‘more than sufficient’ compared to infectious diseases (Table 1). These results were significantly different at p<0.05. Similarly, more than 70% of students reported that they were ‘confident’ or ‘most confident’ in their knowledge of cardiology compared to infectious diseases, where the confidence was only 54%.

A statistically significant relationship was found between sufficiency of education in infectious diseases and confidence in knowledge of infectious diseases only (F(2,110) = 4.49, p = 0.013). Increasing age was not associated with an increase in confidence in knowledge.

### Modes of teaching and confidence in clinical situations

Students rated clinical rotations and informal teaching by residents and registrars as the most useful forms of teaching methods in teaching ID knowledge and prescribing (51.9% and 56.3% of students respectively rated these teaching methods as ‘most useful’), while lectures and attending ward rounds were most likely to be rated as ‘least useful’ (31.3% of students rated each method as ‘least useful’).

**Fig 1:**
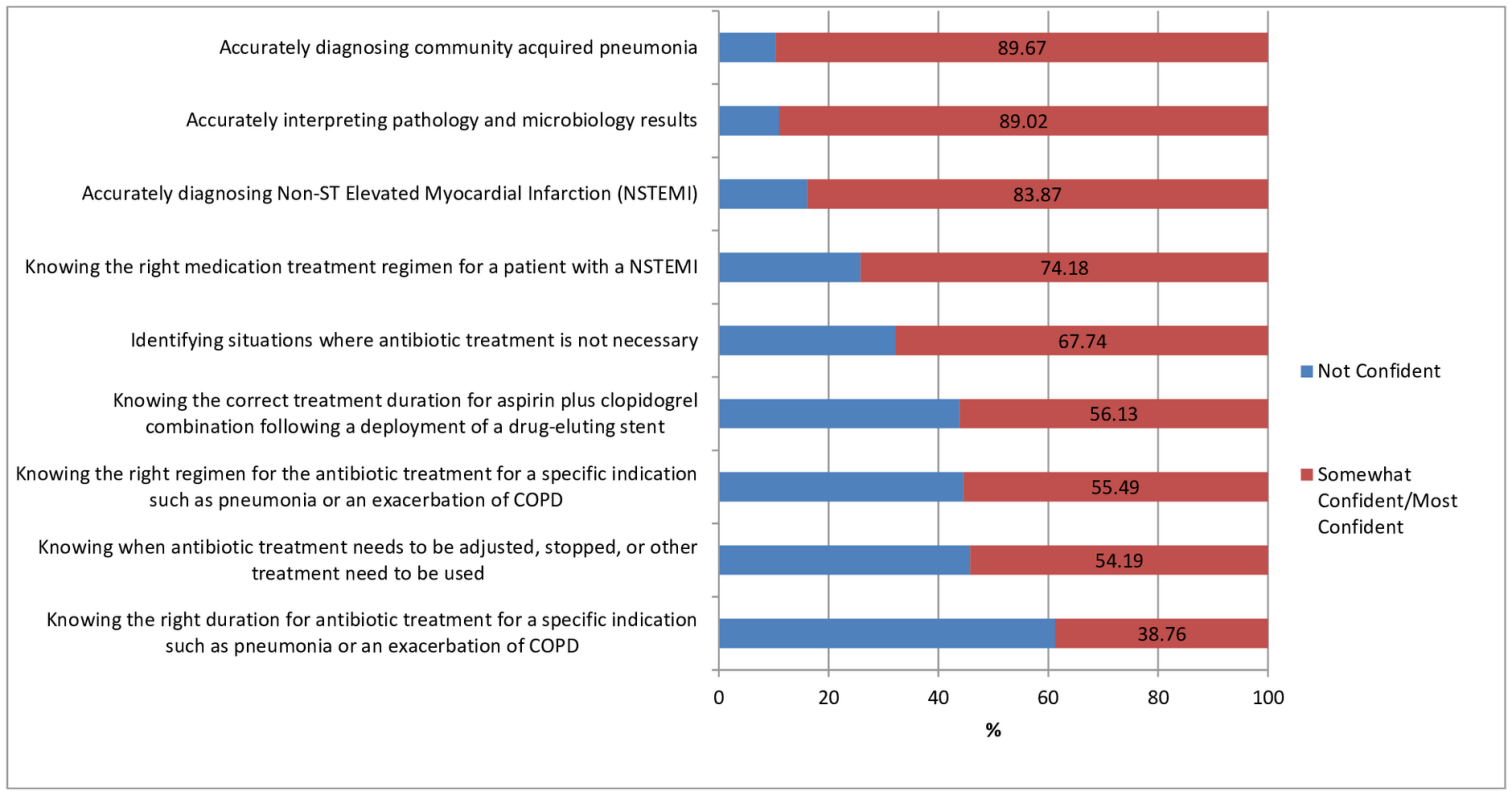
Confidence in knowledge in different clinical situations. NB: Answers were classified as ‘not confident’ if student rated their confidence between -5 (Not at all confident) and 0 (Neutral) on the Likert Scale. Answers were classified as ‘somewhat confident/most confident’ if students rated their confidence as 1 or higher.

Students were most confident in diagnosing community acquired pneumonia and interpreting pathology and microbiology results, and least confident in knowing the right duration for antibiotic treatment (Fig 1).

Males were significantly more likely to be more confident in their knowledge in different clinical situations (U = 2192, p = 0.007), however there was no significant difference between genders on demonstrated clinical knowledge.

### Perceptions of antimicrobial resistance

**Fig 2:**
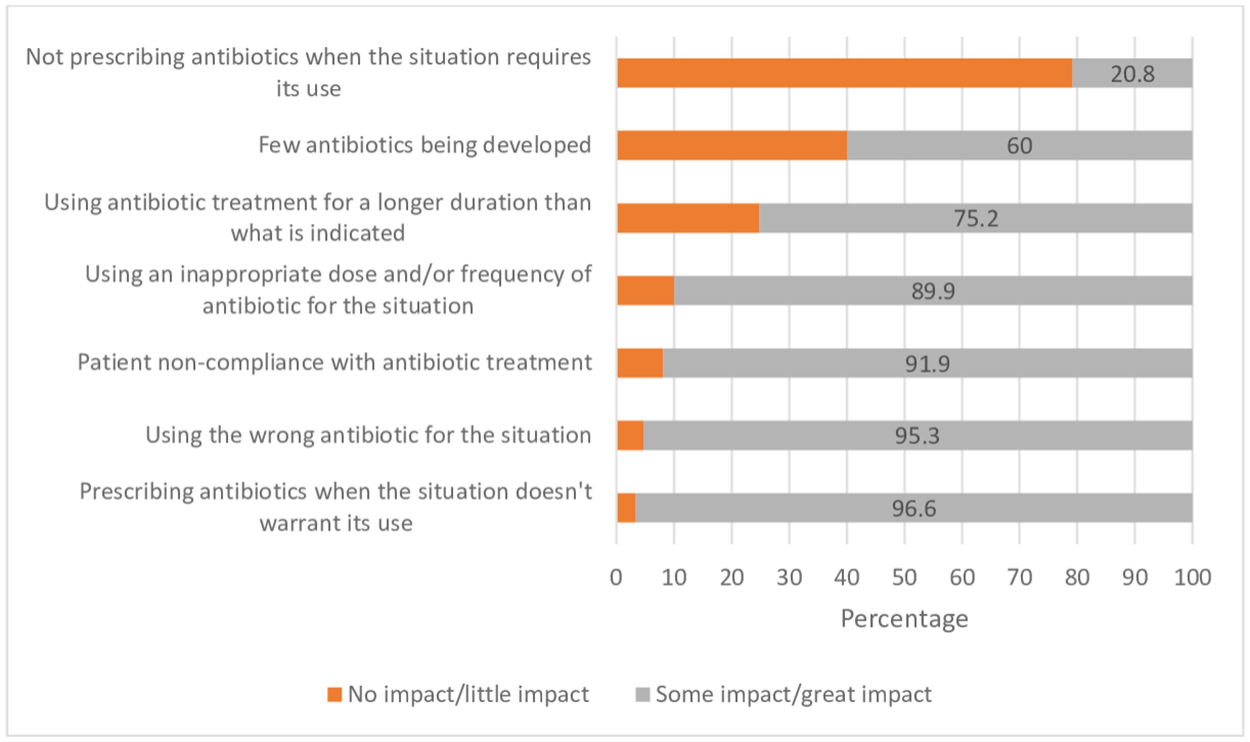
Perceptions of impact different factors have on antimicrobial resistance. NB: Responses were classed as a perception of ‘no impact/little impact’ if students rated between 0 (No Impact) and 4 on the 11 point Likert Scale. Responses were classed as a perception of ‘Some impact/great impact’ if students rated from 5 (Some Impact) to 10 (A Great Impact) on the Likert scale.

Students generally rated most factors as having a great impact on antimicrobial resistance (Fig 2). There was a statistically significant relationship between the total confidence in knowledge in different clinical situations related to antibiotic use and the total score of factors contributing to antimicrobial resistance (sum of each rating of each individual factor) (r = 0.20, p = 0.013).

### Knowledge of prescribing guidelines

Most students (n = 144, 94%) were aware of guidelines available to assist with appropriate antibiotic prescribing, which was a higher proportion compared to students aware of guidelines to assist with management of acute coronary syndrome (n = 132, 86.27%). 56% (n = 86) of students believed that antibiotic guidelines were used in clinical practice at least 50% of the time, and most students (n = 149, 97%) believed that adhering to guidelines was important to reduce the risk of antimicrobial resistance increasing.

### Demonstration of clinical knowledge

A mean score of 52% (SD = 17.62) was obtained for correct answers to the clinical questions overall. Students on average scored better on questions related to knowledge in medication management of cardiovascular diseases (mean 64% SD = 32.56), compared to scores in questions related to antibiotic prescribing (mean 45% SD = 15.75). These results were significantly different at p<0.005.

There was a statistically significant relationship between the percentage of correct answers in cardiology and confidence in clinical cases related to cardiology (r = 0.17, p = 0.03). There was no difference in the demonstration of clinical knowledge between students completing an undergraduate medical degree compared to those completing a graduate degree, which is consistent with other studies finding no difference in clinical performance between students completing these different degrees [20,21].

## Discussion

### Survey

The results of this study has validated the reliability of this survey tool, which is a valuable addition to the medical literature. While previous studies have looked at the confidence of medical students in the area of ID [13–15], no other survey used has been tested for reliability. The significant relationships that were found between the different sections of the survey, including relationships between sufficiency of education and confidence in knowledge in ID and confidence in knowledge in clinical situations overall and demonstrated knowledge in the clinical questions, demonstrate positive relationships and consistency throughout the survey.

The main sections of the survey (sufficiency of education in different subject areas, confidence in knowledge in different subject areas, confidence in knowledge in different clinical situations and perceptions of antibiotic resistance) demonstrated high internal consistency, with Cronbach’s alpha >0.7 in all these sections (S2 Appendix). When analysing item-total statistics for questions investigating confidence in knowledge in different clinical situations, all questions provided Cronbach’s alpha >0.7 if an individual question was deleted, however still produced Cronbach’s alpha results lower than the overall result of the combined questions (with the exception of one question where there was a negligible higher result—see S2 Appendix). This suggests that these questions as a collection support strong reliability in this section. In contrast, the section on perceptions of antibiotic resistance provided variable results on survey reliability if certain questions weren’t included, suggesting that the majority of these questions are required together to ensure valid results from the survey.

The inter-item correlation matrix showed positive values for all sections with the exception of perceptions of antibiotic resistance, where two questions in this section showed negative values from each other (questions 2 and 6 –see S2 Appendix). There was also a higher Cronbach’s alpha value when question 6 was removed compared to the value provided overall, and adjustment of this section of the survey to remove this question could be considered in further studies.

Finally, for questions in all sections apart from those relating to perceptions of antibiotic resistance there were no questions that demonstrated significant correlations, demonstrating independence from each other. For the section on perceptions of antibiotic resistance, a significant correlation was found between question 1 and questions 2 and 6 (S2 Appendix). A higher Cronbach’s alpha was also found if question 1 was removed from this section, suggesting that removing this question if this survey is used for further studies could improve reliability and greater independence of questions in this section.

### Sufficiency of education and effectiveness of teaching methods

The majority of students felt that their education in infectious diseases was ‘not sufficient’ compared to cardiovascular diseases. Similarly, students were more likely to rate their confidence in knowledge lower in infectious diseases compared to the area of cardiology. Studies of medical students overseas have also found that students lacked confidence in their knowledge of appropriate antimicrobial use and would like further education in this area [13–15].

The results of perceptions of the usefulness of different methods of teaching medicines and prescribing in this study are consistent with results found in other studies. Traditional lectures have been found to have a role in teaching basic concepts of subjects such as prescribing, however both medical students and interns have found that clinical placements, informal teaching by registrars, and the opportunity to learn in clinical practice lead to better application of knowledge and greater confidence in medication prescribing [16,22–29].

### Self-reported confidence

Confidence in knowledge in different clinical situations tended to be higher in diagnostic areas (diagnosing community acquired pneumonia [CAP] or Non-ST Elevated Myocardial Infarction [NSTEMI], interpreting pathology and microbiology results, and differentiating between COPD and asthma) compared to appropriately prescribing antimicrobials. These results are consistent with findings internationally that have found medical graduates to be well trained in diagnostics, but less confident in accurately prescribing, particularly in antimicrobial prescribing [15,30–33].

### Knowledge of antimicrobial resistance and practice guidelines

Almost all participants were aware of antibiotic prescribing guidelines available in Australia. This is perhaps in part due to an increasing importance and emphasis placed on medical education on utilising these guidelines to minimise the risk of increasing resistance. Awareness of prescribing guidelines does not automatically translate into utilising these guidelines in clinical practice, however, and all medical practitioners—including graduates and junior doctors—should be adequately supported to have routine access to and make use of guidelines in clinical practice [22,34,35].

Students tended to believe that numerous factors had a high impact on increasing antimicrobial resistance. In particular, ‘prescribing antibiotics when the situation does not warrant its use’, and ‘using the wrong antibiotic for the situation’ were the factors that were more likely to be seen as contributing to antimicrobial resistance. This is consistent with studies conducted in Europe that also found that final year students and interns had a high awareness of the problem of antimicrobial resistance, and the role numerous factors played in contributing to this resistance [15,36].

### Knowledge of ID and antibiotic prescribing

Students had a higher percentage of correct answers to cardiovascular disease related clinical questions compared to those related to infectious diseases and antibiotic use. This suggests that participants had a higher level of clinical knowledge in appropriate medication use in cardiovascular diseases compared to infectious diseases. There was no significant relationship observed between sufficiency of education in infectious diseases or confidence in knowledge of infectious diseases and proportion of correct answers to the clinical questions. This has also been seen in other studies amongst junior doctors that found that confidence in certain areas did not necessarily translate into adequate knowledge and prescribing of particular drugs [34].

### Limitations

The results of this study may be limited by the smaller number of participants, which may not be an accurate representation of all medical students across Australia. This survey was also voluntary, so students who were more academically engaged and interested in this subject matter may have been more likely to complete the survey, potentially limiting the results. While participants in this survey were representative of final year medical students around Australia in the areas of gender, age and the type of degree that was being completed, numbers from larger states were relatively lower when compared to the smaller states. In particular, the number of students from Victoria were limited whereas South Australia and Tasmania had a higher proportion of the respondents, mainly because of the universities’ willingness to participate in the study. Nevertheless, we believe that the presence of a control group and reporting of reliability of the studied scales make this study a useful addition to the existing literature in this space. Additionally, the total number of responses were more than the minimum numbers recommended for a valid reliability analysis [18].

## Conclusions

We reported the results of the first Australian survey of final year medical students assessing their confidence and knowledge in ID and antimicrobial use. This study is the first of its kind to compare knowledge in ID to knowledge of another disease state. We found that not only do Australian medical students feel less confident in their knowledge of ID compared to other conditions but they also demonstrate less clinical knowledge in this area, and these results add to our knowledge in this area of research. Additionally, this study provides a survey tool with demonstrated reliability and validity. Greater emphasis on ID education is urgently needed to improve appropriate antimicrobial use by the junior medical workforce in clinical practice.

## Supporting information

S1 Appendix Survey. (DOCX)

S2 Appendix Reliability analysis and validation of survey tool. (DOCX)

## Abbreviations

CAP: community-acquired pneumonia
COPD: chronic obstructive pulmonary disease
ID: infectious diseases
INR: international normalised ratio
NSTEMI: non-ST segment elevation myocardial infarction
PBL: problem-based learning
SD: standard deviation
STEMI: ST-segment elevation myocardial infarction
