# Supplementary material for: Antimicrobial knowledge and confidence amongst final year medical students in Australia
Source: PLoS One. 2017 Aug 3;12(8):e0182460. doi: 10.1371/journal.pone.0182460 (PMC5542537; doi:10.1371/journal.pone.0182460)
Supplement: S1 Appendix — (DOCX) [file pone.0182460.s001.docx]

**Appendix 1. Survey**

Survey Development

Demographics items measuring age, gender, and proportion of students completing an undergraduate or postgraduate degree were included in the first section. Increasing age of medical students has been associated with increased confidence in knowledge [20], and we were interested in determining if this was the case in our study. While previous studies have found no difference in the clinical performance of students based on whether an undergraduate or graduate medical degree was completed [21,22], we included this item as our study was specific to antimicrobials and this information is lacking in the medical literature.

Section II consists of items about perceived sufficiency of education in ID, confidence in knowledge of ID and relative effectiveness of various teaching modes in acquiring knowledge of ID. Items about the sufficiency of education and confidence in knowledge were included as earlier studies found that medical students would like more education in this area and lacked confidence in their knowledge of appropriate antimicrobial use [13-15]. Items on the relative effectiveness of the mode of teaching were included, as medical students have rated experiential learning highly when compared to traditional class based methods of teaching [27,39,30]. We were particularly keen to know if Australian medical students find a particular teaching method more effective in delivering ID education.

Section III of the survey assessed participant knowledge and attitudes towards prescribing guidelines in clinical practice. Australia has national antimicrobial guidelines available, and many larger organisations will have their own antimicrobial guidelines available based on the national guidelines and local factors [40-42]. Given the multifactorial nature of antimicrobial resistance [43], we included items to assess students’ perceptions and attitudes towards antimicrobial resistance, which was in Section IV. Items on antimicrobial resistance were also motivated by the need to compare results to the findings of European and North American studies where medical students reported that antimicrobial resistance was a national problem, and a major contributor to this resistance was believed to be the overuse of antimicrobials [13,14].

The fifth and final section of the survey involved a series of case-based clinical questions to determine knowledge in selecting the correct treatment in different situations, the right dose and the right duration. Previous studies have assessed confidence in knowledge of antimicrobial use. However, it is unknown how this is related to the demonstration of clinical knowledge in the appropriate selection and prescribing of antimicrobials [13,14]. Answers to the clinical questions were based on national antibiotic prescribing guidelines – Therapeutic Guidelines: Antibiotic, and the Australian Medicines Handbook (AMH) [44,41]. We hypothesised that students who were most confident in their knowledge of infectious diseases would perform better in the clinical questions compared to those that were least confident.

Survey studies measuring medical students’ confidence and knowledge in infectious diseases have lacked control items for comparison [14,15,13]. As such, it is difficult to ascertain whether medical students have a general lack of confidence in their knowledge of therapeutics in general or in infectious diseases in particular. To address this, we included control questions relating to cardiovascular diseases in conjunction with ID related questions.

**Knowledge and Confidence in Appropriate Antibiotic Prescribing Amongst Final Year Medical Students**

**Section I:**

1. Are you:

Male □ Female □

1. Please indicate your age group:

Younger than 25 □ 25-30 years □ 31-35 years □ 36-40 years □

Older than 40 years □

1. Is the medical degree you are completing:

An undergraduate degree □ A graduate degree □

1. Which state are you completing your studies in (for transfer students, please indicate the majority of training)?

ACT □ NSW □ Qld □ SA □ Tas □

WA □ Vic □ Other □

1. FOR GRADUATE MEDICAL PROGRAM STUDENTS ONLY: Please indicate the primary undergraduate qualification:

Pharmacy □ Nursing □ Non-health related □

Other □ please specify …………..

1. Please state the university you are studying at:

………………………..

**Section II: Formal Education and Training on Pharmacology, Prescribing and Infectious Diseases:**

1. Please rate the formal education or training you have received about the following subject matters in your medicine degree and whether you believe it is sufficient for practice. Please consider and recall all modes of education / training such as clinical rotations, lectures, tutorials, Problem Based Learning (PBL) sessions etc.

| **Subject Area** | **Not Sufficient** | **Sufficient** | **More than Sufficient** |
| --- | --- | --- | --- |
| Pharmacology of commonly prescribed drugs [mode of action, dose, adverse effects of medicines etc.] |  |  |  |
| Principles of appropriate and accurate prescription writing [parts of the prescription, legal issues, selecting medicine and dose etc.] |  |  |  |
| Infectious Diseases [common infections such as pneumonia, sepsis, the use of antibiotics, etc.] |  |  |  |
| Cardiovascular Diseases [ischaemic heart disease, heart failure, etc.] |  |  |  |

1. Please rate the relative effectiveness of various modes of delivery in gaining and retaining the knowledge of medicines and prescribing, including but not limited to antibiotics prescribing.

| **Delivery Mode** | **Least Useful** | **Useful** | **Most Useful** |
| --- | --- | --- | --- |
| Lectures |  |  |  |
| Tutorials or workshops of PBL sessions |  |  |  |
| Clinical Rotations |  |  |  |
| Informal teaching by fellow residents and registrars |  |  |  |
| Attending patient care rounds / ward rounds |  |  |  |

1. Please rate your confidence in your knowledge about the following subject matters:

| **Subject Area** | **Somewhat Confident** | **Confident** | **Most Confident** |
| --- | --- | --- | --- |
| Pharmacology [mode of action, dose, adverse effects of medicines, etc.] |  |  |  |
| Principles of prescribing [parts of prescription, legal issues, selecting medicine and dose, etc.] |  |  |  |
| Infectious Diseases [common infections such as pneumonia, sepsis, use of antibiotics, etc.] |  |  |  |
| Cardiovascular Diseases [ischaemic heart diseases, heart failure, etc.] |  |  |  |

**Section III Confidence in Antibiotic Prescribing:**

1. How confident do you feel in the following situations? (please rate your response on the scale, where -5 is not at all confident, 0 is neutral, and 5 is most confident).

- Accurately diagnosing Community Acquired Pneumonia:

Not at all confident Neutral Most confident

-5….….-4……..-3……..-2……..-1……..0……..1……..2……..3………..4………5

- Accurately interpreting pathology and microbiology results:

Not at all confident Neutral Most confident

-5….….-4……..-3……..-2……..-1……..0……..1……..2……..3………..4………5

- Accurately diagnosing Non-ST Elevated Myocardial Infarction (NSTEMI):

Not at all confident Neutral Most confident

-5….….-4……..-3……..-2……..-1……..0……..1……..2……..3………..4………5

- Knowing the right medication treatment regimen for a patient with a NSTEMI:

Not at all confident Neutral Most confident

-5….….-4……..-3……..-2……..-1……..0……..1……..2……..3………..4………5

- Knowing the right regimen [dose, frequency and route of administration] for the antibiotic treatment for a specific indication such as Pneumonia or an exacerbation of COPD:

Not at all confident Neutral Most confident

-5….….-4……..-3……..-2……..-1……..0……..1……..2……..3………..4………5

- Knowing the right duration for antibiotic treatment for a specific indication such as Pneumonia or an exacerbation of COPD:

Not at all confident Neutral Most confident

-5….….-4……..-3……..-2……..-1……..0……..1……..2……..3………..4………5

- Knowing the correct treatment duration for aspirin plus clopidogrel combination following a deployment of a drug-eluting stent:

Not at all confident Neutral Most confident

-5….….-4……..-3……..-2……..-1……..0……..1……..2……..3………..4………5

- Identifying situations where antibiotic treatment is not necessary:

Not at all confident Neutral Most confident

-5….….-4……..-3……..-2……..-1……..0……..1……..2……..3………..4………5

- Knowing when antibiotic treatment needs to be adjusted, stopped, or other treatments need to be used:

Not at all confident Neutral Most confident

-5….….-4……..-3……..-2……..-1……..0……..1……..2……..3………..4………5

**Section IV: Knowledge and Attitude towards Antibiotic Guidelines:**

1. Are you aware of the guidelines available in Australia to assist with appropriate antibiotic prescribing?

Yes □ No □

1. Are you aware of the guidelines available in Australia to assist with appropriate management of acute coronary syndrome?

Yes □ No □

1. From your experience in clinical practice, how often do you believe that antibiotic prescribing guidelines are used to assist with antibiotic prescribing?

Not at all □ Less than 20% of the time □ Between 20-50% of the time □

Between 50-75% of the time □ Between 75-100% of the time □

100% of the time □

1. Do you believe that antibiotic guidelines and adherence to these guidelines is important to reduce the risk of antibiotic resistance increasing?

Yes □ No □

**Section V: Knowledge and Attitude towards Antibiotic Resistance:**

To what extent do you believe the following have an impact on antibiotic resistance developing?

Please rate your response on the scale, where:

1 means no impact

5 means some impact

10 means a great impact

1. Few antibiotics being developed:

No Impact Some Impact A Great Impact 0………1…….2…….3………4……5………6………7………8……….9………10

1. Prescribing antibiotics when the situation doesn’t warrant its use:

No Impact Some Impact A Great Impact 0………1…….2…….3………4……5………6………7………8……….9………10

1. Using the wrong antibiotic for the situation:

No Impact Some Impact A Great Impact 0………1…….2…….3………4……5………6………7………8……….9………10

1. Using an inappropriate dose and / or frequency of antibiotic for the situation:

No Impact Some Impact A Great Impact 0………1…….2…….3………4……5………6………7………8……….9………10

1. Using antibiotic treatment for a longer duration than what is indicated:

No Impact Some Impact A Great Impact 0………1…….2…….3………4……5………6………7………8……….9………10

1. Not prescribing antibiotics when the situation requires its use:

No Impact Some Impact A Great Impact 0………1…….2…….3………4……5………6………7………8……….9………10

1. Patient non-compliance with antibiotic treatment (such as not taking it as prescribed, not completing the course, or taking too much):

No Impact Some Impact A Great Impact 0………1…….2…….3………4……5………6………7………8……….9………10

**Section VI: Clinical Cases**

**Clinical Case 1:**

The following questions relate to this case: (answers in **bold** are the correct answers)

Anna is a 39 year old female who has mild COPD that is managed with prn salbutamol MDI. She has no allergies, and is not pregnant or breastfeeding. She has presented to her doctor with pain and stinging when urinating and symptoms of urgency. She is subsequently diagnosed with an acute uncomplicated urinary tract infection, and her doctor decides to prescribe her trimethoprim 300mg daily.

1. How long should this treatment continue?
2. 1 day
3. **3 days**
4. 7 days
5. 14 days
6. What would your treatment recommendation be if Anna reported that she returned three weeks ago from living in India for the past two months?
7. Trimethoprim 300mg daily for 7 days
8. Trimethoprim plus sulfamethoxazole 160/800mg twice daily for 7 days
9. **Nitrofurantoin 100mg twice daily for 5 days**
10. Amoxycillin 500mg four times a day for 5 days
11. Anna was treated with the recommended treatment above, however over the next two days her condition deteriorated, she developed a fever, and became increasingly unwell. She was diagnosed as having sepsis and was admitted to hospital. What would the most appropriate treatment be at this time?
12. **IV ceftriaxone 2g daily plus IV azithromycin 500mg daily plus IV metronidazole 500mg twice daily**
13. IV benzylpenicillin 1.2g daily plus oral nitrofurantoin 100mg four times daily
14. Oral amoxycillin/clavulanic acid 875mg/125mg twice daily plus oral doxycycline 100mg twice daily
15. IV cefotaxime 2g three times daily plus oral azithromycin 500mg daily

**Clinical Case 2:**

The following questions relate to this case: (answers in **bold** are the correct answers)

Sophie is a 2 year old girl with a weight of 12kg who has presented with ear pain in her left ear, which started the night before. She is healthy apart from a history of asthma, is still able to play and eat, and has not allergies to medications. Upon examination you diagnose her with acute otitis media.

1. What is the most appropriate initial course of treatment?
2. Begin treatment with amoxycillin 300mg every eight hours
3. **Provide symptomatic treatment with paracetamol, and advise the parents to return for review if symptoms are still present after 2 days**
4. Begin treatment with amoxycillin + clavulanic acid
5. Refer her to a specialist as it is unusual for children her age to have ear infections
6. 2 days later Sophie returns for review. Her ear pain is no better, and she is feeling tired and irritated as she hasn’t been able to sleep for the past few days. What would your treatment recommendation be?
7. Treat with amoxycillin 360mg every six hours
8. Provide symptomatic treatment with paracetamol, and reassure the parents that her condition will improve in the next few days
9. **Treat with amoxycillin 180mg every eight hours**
10. Refer her to a specialist as there is no further treatment that can be provided at a general level
11. How long should antibiotic treatment continue for?
12. 2 days
13. **5 days**
14. 7 days
15. 10 days

**Clinical Case 3:**

The following questions relate to this case: (answers in **bold** are the correct answers)

A 45 year old female patient presents with suspected community acquired pneumonia. She is healthy otherwise apart from experiencing occasional migraines, has no documented drug allergies, and her only other medications include levonorgestrel 150mcg/ethinyloestradiol 30mcg (Levlen ED) 1 tablet daily and paracetamol 500mg/codeine 30mg (Panadeine Forte) 1-2 tablets qid prn. Upon examination her respiratory rate is 35 breaths per minute, her blood pressure reading is 135/85mmHg, and her heart rate is 80 beats per minute.

1. What would be your initial course of action?
2. **Admit the patient to hospital and prescribe IV benzylpenicillin 1.2g every six hours and oral doxycycline 100mg twice daily**
3. Prescribe oral amoxycillin 1 gram every eight hours plus oral doxycycline 100mg twice daily
4. Advise symptomatic treatment only for this patient and recommend she seek medical attention if symptoms do no improve within 2 days
5. Prescribe oral amoxycillin 1 gram every eight hours
6. What would your treatment recommendation be if the patient had a respiratory rate of 35 breaths per minute, a blood pressure reading of 110/85mmHg, a heart rate of 90 beats per minute and an oxygen saturation of 85%, and had a history of nausea and diarrhoea with phenoxymethylpenicillin in the past?
7. Oral amoxycillin 1 gram every eight hours plus oral doxycycline 100mg twice daily
8. **IV benzylpenicillin 1.2 grams every six hours plus oral doxycycline 100mg twice daily**
9. IV ceftriaxone 1 gram daily plus oral doxycycline 100mg twice daily
10. Oral moxifloxacin 400mg daily
11. What would your recommendation for treatment be if the patient lived in a tropical area in Australia and it is the wet season?
12. **Prescribe the same antibiotic treatment**
13. Add in IV azithromycin
14. Prescribe IV ceftriaxone plus IV gentamicin
15. Prescribe IV meropenem plus IV azithromycin
16. While this patient was waiting for her blood culture results to come through, she began to complain of severe sudden onset chest pain. An ECG reading revealed that there was an ST segment elevation and she subsequently received a STEMI diagnosis.

After initial management what ongoing antiplatelet treatment should this patient receive?

1. Aspirin 300mg daily
2. **Aspirin 150mg daily plus clopidogrel 75mg daily**
3. Enoxaparin SC 100mg twice daily
4. Ticagrelor 90mg twice daily

**Clinical Case 4:**

The following questions relate to this case: (answers in **bold** are the correct answers)

Alan is a 61 year old male with a medical history of asthma and chronic obstructive pulmonary disease (COPD), as well as hypertension, hyperlipidaemia, and osteoarthritis. His current medications are:

- Salbutamol 100mcg MDI 1-2 puffs q4h prn
- Tiotropium 18mcg 1 capsule inhaled daily
- Candesartan/Hydrochlorothiazide 16mg/12.5mg 1 tablet in the morning
- Rosuvastatin 10mg 1 tablet in the evening
- Paracetamol slow release 665mg 2 tablets tds

Despite these medical conditions, Alan usually feels pretty healthy, and is able to do all his daily activities with ease. However, over the last three days Alan has become increasingly breathless with any extra exertion, has been unable to complete his usual daily walk around the block, and is finding he is breathing much faster than usual. He is using his salbutamol inhaler several times each hour, and is not getting relief like he usually does. He presents to the hospital’s emergency department and is admitted to the ward for further management and review. This is the first exacerbation that Alan has had.

1. What would be the initial course of action?
2. Take a sputum culture to determine whether there is an infectious cause to the exacerbation
3. Take a spirometry reading
4. Test the patient’s blood sugar levels and treat to control these if required
5. **Take an accurate medical history including symptoms, comorbidities and adherence to medications**
6. It was found that Alan had increasing sputum purulence, and antibiotics were prescribed. What would be the treatment of choice for Alan?
7. Amoxycillin/clavulanic acid 875mg/125mg 1 tablet bd for 5 days
8. Azithromycin 500mg daily for 3 days
9. **Amoxycillin 500mg tds for 5 days**
10. Cefuroxime 500mg bd for 5 days
11. While Alan is in hospital, there becomes a worldwide shortage of rosuvastatin and the hospital is unable to source it anywhere. A decision is made to prescribe simvastatin instead. At what time of day will it have its maximum efficacy?
12. In the morning
13. **In the evening**
14. It should be taken twice a day
15. It doesn’t matter what time of day it is taken – it will still have the same efficacy
16. After one week Alan is feeling much better and a decision is made to discharge him home from hospital. What would not be a recommended medication treatment for Alan at this stage?
17. **Cephalexin 250mg daily ongoing**
18. Salbutamol 100mcg MDI 1-2 puffs prn
19. Tiotropium 18mcg 1 capsule inhaled daily
20. Fluticasone/Salmeterol 125mcg/25mcg MDI 2 puffs bd

**Clinical Case 5:**

The following questions relate to this case: (answers in **bold** are the correct answers)

David is a 25 year old male of Aboriginal origin. He is currently living in an Aboriginal community in the Northern Territory with his extended family, and lives a basic lifestyle that includes cooking over camp fires, bathing in the nearby river, and sleeping in makeshift tents. He is usually healthy, takes no regular medications, and has no known medication allergies. Several months ago, David had a severe illness where he was running a high fever and had pain in multiple joints. He was taken to a local hospital where he was treated symptomatically, and once recovered, returned to his home. He has presented to the hospital again with the same symptoms, and following recovery from this second episode he is experiencing ongoing fatigue and dyspnoea. Following further examination he is diagnosed with rheumatic heart disease.

1. With continued monitoring, David developed atrial fibrillation. What would be the initial choice of treatment if the goal is to obtain rate control?
2. Verapamil SR 160mg daily
3. Diltiazem SR 360mg daily
4. **Metoprolol 25mg bd**
5. Amiodarone 200mg tds
6. Several years have passed and David is managing well. However, he had recently been to the dentist and requires a tooth to be extracted. What would be the recommended prophylactic antibiotic treatment in this case?
7. Amoxycillin 2 grams orally 1 hour before the procedure, then 500mg tds for five days
8. **Amoxycillin 2 grams orally 1 hour before the procedure**
9. Amoxycillin 500mg tds for five days following the procedure
10. The patient doesn’t require prophylactic antibiotics in this case
11. What would your recommendation be if David reported that he experienced an urticarial and bronchospasm reaction to benzylpenicillin in the past?
12. Amoxycillin 2 grams orally 1 hour before the procedure
13. Cephalexin 2 grams orally 1 hour before the procedure
14. **Clindamycin 600mg orally 1 hour before the procedure**
15. The patient should not be given prophylactic antibiotics in this case
16. What would your treatment recommendation be if David was having impressions and construction of dentures done?
17. Amoxycillin 2 grams orally 1 hour before the procedure, then 500mg tds for 5 days
18. Amoxycillin 2 grams orally 1 hour before the procedure
19. Amoxycillin 500mg tds for five days following the procedure
20. **The patient doesn’t require prophylactic antibiotics in this case**

**Clinical Case 6**

The following questions relate to this case: (answers in **bold** are the correct answers)

Margaret is a 55 year old woman who has presented complaining of diarrhoea for the past three days. When asked about her medical history she says that she takes metformin 500mg daily for type II diabetes and warfarin at night for a deep vein thrombosis. She is looking well, with no signs of dehydration, and states that she is just sick of going to the toilet all the time.

1. What would be important questions to ask Margaret?
2. Whether she is passing blood or pus in the stool
3. If she has recently travelled overseas
4. If she has recently had treatment with antibiotics
5. **These are all important questions to ask**
6. Margaret reveals that she has recently returned home from Vietnam, and since she has been home she has been feeling nauseous and unwell, and had felt feverish for several days prior to the diarrhoea. What would be the most appropriate management of Margaret?
7. Ciprofloxacin 500mg orally bd for three days
8. **Azithromycin 500mg orally daily for three days**
9. Amoxycillin / clavulanic acid 875mg/125mg orally bd for five days
10. Antibiotic treatment is not indicated
11. What would your treatment recommendation be if Margaret had not reported any recent overseas travel?
12. **Ciprofloxacin 500mg orally bd for three days**
13. Azithromycin 500mg orally daily for three days
14. Amoxycillin / Clavulanic acid 875mg/125mg orally bd for five days
15. Antibiotic treatment is not indicated
16. As Margaret is also currently taking warfarin what other advice would you give her at this stage?
17. As she is currently unwell, she should stop taking warfarin and only recommence it once she is feeling better
18. **She will require regular monitoring of her INR, as being unwell may affect her INR result and she may require dosage adjustments during this time**
19. She should not take her warfarin at the same time as taking antibiotics as if she gets nauseous with the antibiotic the warfarin won’t be absorbed as well
20. All antibiotics can decrease the INR so the warfarin dose should be doubled during treatment

**Further Comments**

Please use the following space to provide any further comments, suggestions or opinions about the subject matter:

…………………………………………………………………………………………………………………………………………………………………………………………………………………………………………………………………………………………………………………………………………………………………………………………………………………………………………………………………….

Thank you for your participation
