# Supplementary material for: Antimicrobial knowledge and confidence amongst final year medical students in Australia
Source: PLoS One. 2017 Aug 3;12(8):e0182460. doi: 10.1371/journal.pone.0182460 (PMC5542537; doi:10.1371/journal.pone.0182460)
Supplement: S2 Appendix — (DOCX) [file pone.0182460.s002.docx]

**Appendix 2. Reliability analysis and validation of survey tool**

| **Survey Section** | **Cronbach’s alpha** |
| --- | --- |
| Sufficiency of education in different subject areas | 0.807 |
| Confidence in knowledge in different subject areas | 0.805 |
| Confidence in knowledge in different clinical situations | 0.868 |
| Perceptions of antibiotic resistance | 0.717 |

**Table 2.1: Cronbach’s alpha calculated from different survey sections**

| **Item-Total Statistics - Sufficiency of Education** | | | | | |
| --- | --- | --- | --- | --- | --- |
|  | Scale Mean if Item Deleted | Scale Variance if Item Deleted | Corrected Item-Total Correlation | Squared Multiple Correlation | Cronbach's Alpha if Item Deleted |
| Infectious Diseases | 4.2500 | 1.132 | .470 | .249 | .922 |
| Cardiovascular Diseases | 3.9375 | .927 | .803 | .760 | .582 |
|  |  |  |  |  |  |

| **Inter-Item Correlation Matrix – Sufficiency of Education**   \|  \| Infectious Diseases \| Cardiovascular Diseases \| \| --- \| --- \| --- \| \| Infectious Diseases \| 1.000 \| .498 \| \| Cardiovascular Diseases \| .498 \| 1.000 \| \|  \|  \|  \| |
| --- | --- | --- | --- | --- | --- | --- | --- | --- | --- | --- | --- | --- |

| **Item-Total Statistics – Confidence in Knowledge** | | | | | |
| --- | --- | --- | --- | --- | --- |
|  | Scale Mean if Item Deleted | Scale Variance if Item Deleted | Corrected Item-Total Correlation | Squared Multiple Correlation | Cronbach's Alpha if Item Deleted |
| Infectious Diseases | 3.7736 | 1.531 | .465 | .218 | .910 |
| Cardiovascular Diseases | 3.4780 | 1.162 | .749 | .700 | .627 |
|  |  |  |  |  |  |

**Inter-Item Correlation Matrix – Confidence in Knowledge**

|  | Infectious Diseases | Cardiovascular Diseases |
| --- | --- | --- |
| Infectious Diseases | 1.000 | .433 |
| Cardiovascular Diseases | .433 | 1.000 |
|  |  |  |

| **Inter-Item Correlation Matrix – Confidence in Clinical Situations** | | | | | | | | | |
| --- | --- | --- | --- | --- | --- | --- | --- | --- | --- |
|  | A4 | B4 | D4 | E4 | F4 | G4 | H4 | I4 | J4 |
| A4 | 1.000 | .700 | .596 | .544 | .457 | .318 | .297 | .521 | .438 |
| B4 | .700 | 1.000 | .522 | .473 | .468 | .358 | .290 | .458 | .400 |
| D4 | .596 | .522 | 1.000 | .719 | .362 | .288 | .333 | .382 | .296 |
| E4 | .544 | .473 | .719 | 1.000 | .528 | .415 | .408 | .327 | .322 |
| F4 | .457 | .468 | .362 | .528 | 1.000 | .736 | .424 | .407 | .448 |
| G4 | .318 | .358 | .288 | .415 | .736 | 1.000 | .449 | .431 | .498 |
| H4 | .297 | .290 | .333 | .408 | .424 | .449 | 1.000 | .259 | .310 |
| I4 | .521 | .458 | .382 | .327 | .407 | .431 | .259 | 1.000 | .709 |
| J4 | .438 | .400 | .296 | .322 | .448 | .498 | .310 | .709 | 1.000 |

| **Item-Total Statistics – Confidence in Clinical Situations** | | | | | |
| --- | --- | --- | --- | --- | --- |
|  | Scale Mean if Item Deleted | Scale Variance if Item Deleted | Corrected Item-Total Correlation | Squared Multiple Correlation | Cronbach's Alpha if Item Deleted |
| A4 | 9.1613 | 169.993 | .660 | .613 | .851 |
| B4 | 9.2065 | 169.814 | .623 | .535 | .853 |
| C4 | 9.2323 | 166.907 | .588 | .599 | .855 |
| D4 | 9.9290 | 163.599 | .648 | .613 | .850 |
| E4 | 11.1677 | 153.985 | .688 | .632 | .845 |
| F4 | 12.0387 | 155.583 | .634 | .605 | .850 |
| G4 | 11.1871 | 158.387 | .481 | .272 | .870 |
| H4 | 10.3677 | 163.546 | .604 | .572 | .853 |
| I4 | 11.2323 | 160.426 | .598 | .557 | .854 |

**NB: A4: Accurately diagnosing Community Acquired Pneumonia**

**B4: Accurately interpreting pathology and microbiology results**

**C4: Accurately diagnosing Non-ST Elevated Myocardial Infarction (NSTEMI)**

**D4: Knowing the right medication treatment regimen for a patient with a NSTEMI**

**E4: Knowing the right regimen [dose, frequency and route of administration] for the antibiotic treatment for a specific indication such as Pneumonia or an exacerbation of COPD**

**F4: Knowing the right duration for antibiotic treatment for a specific indication such as Pneumonia or an exacerbation of COPD**

**G4: Knowing the correct treatment duration for aspirin plus clopidogrel combination following a deployment of a drug-eluting stent**

**H4: Identifying situations where antibiotic treatment is not necessary**

**I4: Knowing when antibiotic treatment needs to be adjusted, stopped, or other treatments need to be used**

| **Item-Total Statistics – Perceptions of Antibiotic Resistance** | | | | | |
| --- | --- | --- | --- | --- | --- |
|  | Scale Mean if Item Deleted | Scale Variance if Item Deleted | Corrected Item-Total Correlation | Squared Multiple Correlation | Cronbach's Alpha if Item Deleted |
| Resistance1 | 40.8255 | 65.294 | .258 | .090 | .740 |
| Resistance2 | 37.6040 | 65.308 | .532 | .549 | .665 |
| Resistance3 | 38.1879 | 62.086 | .621 | .616 | .644 |
| Resistance4 | 38.6577 | 59.929 | .611 | .521 | .640 |
| Resistance5 | 40.1879 | 61.586 | .461 | .281 | .676 |
| Resistance6 | 43.1275 | 72.180 | .194 | .159 | .739 |
| Resistance7 | 38.5369 | 64.034 | .449 | .292 | .680 |

**NB: Resistance 1: Few antibiotics being developed**

**Resistance 2: Prescribing antibiotics when the situation doesn’t warrant its use**

**Resistance 3: Using the wrong antibiotic for the situation**

**Resistance 4: Using an inappropriate dose and / or frequency of antibiotic for the situation**

**Resistance 5: Using antibiotic treatment for a longer duration than what is indicated**

**Resistance 6: Not prescribing antibiotics when the situation requires its use**

**Resistance 7: Patient non-compliance with antibiotic treatment (such as not taking it as prescribed, not completing the course, or taking too much)**

| **Inter-Item Correlation Matrix – Perceptions of Antibiotic Resistance** | | | | | | | |
| --- | --- | --- | --- | --- | --- | --- | --- |
|  | Resistance1 | Resistance2 | Resistance3 | Resistance4 | Resistance5 | Resistance6 | Resistance7 |
| Resistance1 | 1.000 | .184* | .154 | .129 | .231 | .162* | .160 |
| Resistance2 | .184* | 1.000 | .704 | .538 | .286 | -.075 | .425 |
| Resistance3 | .154 | .704 | 1.000 | .663 | .373 | .064 | .415 |
| Resistance4 | .129 | .538 | .663 | 1.000 | .421 | .120 | .465 |
| Resistance5 | .231 | .286 | .373 | .421 | 1.000 | .286 | .158 |
| Resistance6 | .162* | -.075 | .064 | .120 | .286 | 1.000 | .153 |
| Resistance7 | .160 | .425 | .415 | .465 | .158 | .153 | 1.000 |

**Table 2.2: Item total statistics and inter-item correlation matrix**

***Significant correlation shown at p<0.05**
